# Economic recession and cardiovascular disease among women: a cohort study from Eastern Finland

**DOI:** 10.1177/1403494821990259

**Published:** 2021-02-10

**Authors:** Rand Jarroch, Tomi-Pekka Tuomainen, Behnam Tajik, Jussi Kauhanen

**Affiliations:** Institute of Public Health and Clinical Nutrition, University of Eastern Finland, Kuopio, Finland

**Keywords:** Economic recession, cardiovascular disease, women, population-based, cohort study

## Abstract

**Aims:**

Little is known about the effect of economic recessions on cardiovascular disease. Therefore, we investigated the association of the economic recession in Finland in the 1990s with the incidence of cardiovascular disease among middle-aged and older women.

**Methods:**

A total of 918 women aged 53–73 years were examined for health and socioeconomic position in 1998–2001, as part of the population-based prospective Kuopio Ischaemic Heart Disease Risk Factor Study. The participants were asked whether Finland’s economic recession in the early 1990s had affected their lives socially or economically. The cohort was followed for 18 years, and incident physician-diagnosed cases of cardiovascular disease were obtained through record linkage with the national hospital discharge registry that covers every hospitalisation in Finland. Cox proportional hazards regression models were used to estimate the risk of cardiovascular disease among those with and without exposure to socioeconomic hardships during the recession, after adjusting for possible confounders.

**Results:**

At the baseline, 587 women reported having experienced socioeconomic hardships due to the recession. During the 20 years’ follow-up, 501 women developed cardiovascular disease. After adjustment for age, the risk of cardiovascular disease was 27% higher among women exposed to socioeconomic hardships compared to those who were not (hazard ratio 1.27, 95% confidence interval 1.06–1.53, *P*=0.012). Further adjustments for overall socioeconomic position at baseline, prior cardiovascular health, and lifestyle factors did not attenuate the association (hazard ratio 1.23, 95% confidence interval 1.02–1.5, *P*=0.029).

**Conclusions:**

The early 1990s economic recession was associated with a subsequently increased risk of cardiovascular disease among Finnish women.

## Introduction

Economic recessions may cause dramatic and sudden changes in socioeconomic position (SEP) in some part of the general population [1, 2]. The subsequent disadvantages are termed socioeconomic hardships, and they may affect social or financial domains in individuals’ lives at home or work [3]. Although there is a well-established literature on the role of SEP in cardiovascular disease (CVD) development during the normal life cycle, a limited number of studies have investigated the role of economic recessions in CVD morbidity and mortality. Findings from prior studies are controversial and inconclusive. Few studies have suggested that recessions are associated with a higher risk of incident CVD in the future [1] and increased CVD mortality rates during the recession period itself [4–6]. Other studies, however, have argued that CVD mortality may even decrease during recessions [7–10]. Remarkably, most of those studies focused only on unemployment to measure the hardships caused by recessions [4–10].

Finland fell into an exceptional economic decline between 1991 and 1994. The recession effects were felt for several years and led to long-lasting socioeconomic hardships for many in the Finnish population [11]. Few studies have investigated the possible impact of the recession on cardiovascular health. However, those studies covered too short a period of time after the recession, while a longer follow-up was needed [2] because the recession’s physiological adverse events tend to appear many years later [12].

Women are generally under-represented in epidemiological studies on CVD, although this gender bias has somewhat changed during the past two decades when gender-specific pathways in CVD aetiology have been better understood. Some studies have suggested that the SEP gradient may be even steeper in predicting the risk of CVD among women than among men [13]. Moreover, studies on longer-term effects of recessions have shown different CVD patterns between the genders. Women, in contrast to men, had higher rates of hypertension during the economic recovery, rather than during the recession period itself [1]. Therefore, the aim of our study was to assess how socioeconomic hardships resulting from the 1990s economic recession are associated with the long-term incidence of CVD in a population-based sample of middle-aged and older women in Eastern Finland.

## Methods

### Study population

We performed a prospective analysis among participants from the Kuopio Ischaemic Heart Disease Risk Factor Study (KIHD) [14]. KIHD is an ongoing prospective population-based study that aims to investigate the different risk factors of CVD and other non-communicable disease outcomes among middle-aged men and women in the Kuopio region in Eastern Finland [15]. A total of 920 women (78.4% of the 1173 eligible) aged 53–73 years participated in the study and were examined between 1998 and 2001.

The KIHD protocol was approved by the research ethics committee of the University of Kuopio and complies with the Declaration of Helsinki. All subjects signed written informed consents.

**Figure 1.**
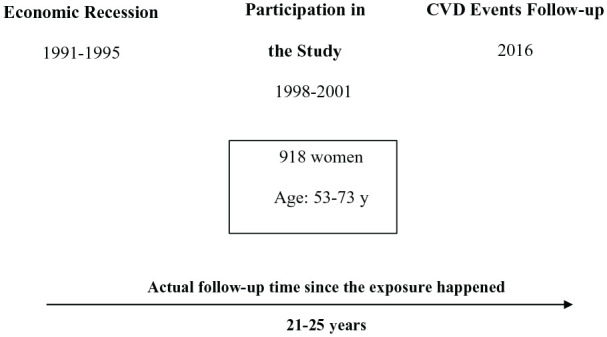
Study timeline.

After excluding participants with missing data on experiencing socioeconomic hardships (*n*=2), a total of 918 women were included in the study. Two baselines were used in this study. The first was when the women participated in the study between 1998 and 2001. Participants were asked to recall retrospectively how they experienced the economic recession between 1991 and 1995 (the actual baseline) (Figure 1).

### Measurements

#### Ascertainment of CVD follow-up events

Incident CVD was defined by record linkage from the national computerised hospitalisation registry that covers every hospitalisation in Finland. CVD during the follow-up was coded according to the International Classification of Disease, version 10 (ICD-10) codes (CVD I00-99) [16] assigned by a physician in secondary or tertiary care. If a subject had multiple non-fatal coronary events during the follow-up, the first event after the baseline was defined as the outcome event. All CVD events that occurred by the end of 2016 were included, and the actual follow-up time extended to more than 20 years.

#### Baseline SEP

SEP refers to the social position of the individual compared to other individuals within society. It indicates both the resource-based and prestige-based measures in childhood and adulthood [17]. In our study, participants completed very detailed questionnaires on their socioeconomic background, describing their possessions, income, debt, investments, residence, education, occupation, marital status, etc.

To adjust for adulthood SEP at baseline, we only used education and marital status because the other frequently used SEP variables are already implemented in the participants’ response about their economic recession experience. Besides, education was not affected by the recession, and it is generally considered one of the best measures of social position in Finland, also among women.

#### Defining socioeconomic hardships

Participants in our study were asked whether Finland’s economic recession in 1991–1994, which peaked 4–9 years prior to the baseline examination, had influenced their personal or family economic situation. Detailed questions on income reduction, unemployment, bankruptcy and loss of property were included. Original responses were grouped into two categories: women who did and women who did not experience any socioeconomic hardships because of the recession, regardless of the number of hardships experienced.

#### Other risk factors

Marital status was divided into four groups: married or living with a partner, not married, separated or divorced and widowed. Smoking status was determined in the baseline questionnaire [15], and alcohol consumption was assessed using the Nordic alcohol consumption inventory for drinking behaviour over the previous 12 months with a structured quantity-frequency method [18]. A trained nurse checked and completed the questionnaire during an interview. Physical activity was assessed using the 12-month physical activity questionnaire to record the duration of the most common physical activities of Finnish middle-aged people [19]. Dietary intake of healthy nutrients (fruits, vegetables and berries) was calculated based on 4-day food records [20].

The criteria for hypertension were: systolic/diastolic blood pressure greater than 140/90 mmHg (average of five measures at baseline visit), clinical diagnosis of hypertension, or use of antihypertensive medication. Type 2 diabetes (T2D) was defined as a self-reported physician diagnosis of T2D and/or fasting plasma glucose of 7.0 mmol/L or greater. The history of CVD was defined by a physician at the baseline examination as having a diagnosis of CVD other than hypertension. The CVD (I00-99) diagnosis report included the diagnosis of coronary heart disease (CHD) (I20-25), and/or cardiomyopathy (I42-43), heart failure (HF) (I50, I11.0), stroke (I60-64), arteriosclerosis (I70.0-70.2, I70.8-70.9) and/or other heart disease, in addition to a previously diagnosed ischaemic heart disease (IHD) (I20-25).

#### Statistical analysis

The distribution of baseline socioeconomic, lifestyle and clinical characteristics were examined in the recession-time hardship categories (yes/no) by means and analysis of variance for continuous variables and chi^2^ independency test for categorical variables to explore bivariate relationships.

Cox regression models were used to estimate hazards ratios (HRs) for the risk of CVD over the hardship categories. Those who did not experience any hardships were the reference group. The validity of the proportional hazard assumption was satisfied when evaluating the Schoenfeld residuals. The confounders in the multivariate models were selected based on established risk factors for CVD or on associations with exposures or outcomes in the present analysis.

Seven hierarchical models were adjusted for potential confounders in the prospective analyses. Each of the lifestyle factors was examined in a separate model to investigate its impact on CVD development. Model 1 was adjusted only for age. Model 2 was adjusted for the sociodemographic variables of education (years) and marital status (married or living as a couple, not married, separated or divorced, widowed). Models 3, 4, 5 and 6 were adjusted for smoking status (yes/no), alcohol intake (g/week), total duration of leisure time physical activity (hours/year) and the mean consumption of vegetables, fruits and berries in 4 days (g), respectively. Finally, in model 7, we adjusted for the history of CVD morbidity at baseline (yes/no), which was positive if one or more of the following criteria was met: prior history of IHD, diagnosed hypertension, or history of diabetes.

The cohort mean was used to replace missing values within each of the covariates (<0.5%). All *P* values were two-sided (α=0.05). All analyses were performed with the SPSS statistical software (version 25; SPSS Inc., Chicago, IL, USA).

## Results

### Baseline characteristics

A total of 587 (64%) women reported experiencing socioeconomic hardships during the preceding recession. The baseline characteristics of the participants, by exposure category, are presented in Table I. Women who experienced hardships due to the recession were more likely to be younger and more likely to smoke as compared to women who did not experience any hardships. The exposed group also had on average a higher body mass index (BMI) and lower systolic blood pressure. It is also worth noting that the women in both groups were on average already unemployed before the recession happened.

**Table I.** Baseline characteristics according to the level of socioeconomic hardships caused by the 1990s economic recession.

Level of the socioeconomic hardships due to the economic recession			
Variables	Did not experience socioeconomic hardships (*n*=331)	Experienced socioeconomic hardships (*n*=587)	*P* value
Age (years)	65.1 (6.2)	62.1 (6.4)	⩽0.001
Education (years)	9.7 (3.5)	9.7 (3.2)	0.87
Income (€/year)	13,848 (7739)	13,496 (7887)	0.51
Marital status			0.49
Married/living as a couple	67.7%	64.2%	
Not married	8.8%	8.2%	
Divorced/separated	7.9%	14%	
Widowed	15.7%	13.6%	
Unemployment year	1989 (10)	1987 (16)	0.12
Current smoker (%)	5.1%	9.9 %	0.012
Alcohol intake (g/w)	19.3 (38.2)	18.6 (37.2)	0.8
BMI (kg/m^2^)	27.7 (4.8)	28.7 (5.2)	0.006
Physical activity (hours/year)	601.5 (482.9)	631.3 (544.6)	0.4
Total cholesterol (mmol/l)	5.7 (0.90)	5.7 (0.93)	0.94
Serum LDL (mmol/l)	3.7 (0.88)	3.7 (0.94)	0.92
Serum HDL (mmol/l)	1.35 (0.35)	1.34 (0.29)	0.71
Serum TG (mmol/l)	1.25 (0.62)	1.24 (0.67)	0.8
Treated hypertension (%)	65.9%	66.1%	0.94
Systolic blood pressure (mmHg)	139.2 (17.7)	136.3 (16.8)	0.014
Diastolic blood pressure (mmHg)	80.3 (8.4)	80.4 (8.6)	0.86
Diabetes (%)	12.4%	9.2%	0.12
CRP (mg/L)	3.0 (4.68)	3.1 (3.1)	0.9

Results being presented are mean ± SD for continuous variables and *n* (%) for categorical data.

BMI: body mass index; LDL: low-density lipoprotein; HDL: high-density lipoprotein; TG: triglycerides; CRP: C-reactive protein.

The mean ± standard deviation (SD) age for women with socioeconomic hardships was 62.1±6.4 years compared to 65.1±6.2 years for those who reported none (*P*<0.001). The percentage of smoking in those two categories was 9.9% and 5.1%, respectively (*P*=0.012). BMI was 28.7±5.2 and 27.7±4.8, respectively (*P*<0.01). The systolic blood pressure among those who experienced hardships was 136.3±16.8 mmHg compared to 139.2±17.7 mmHg in the group that did not (*P*=0.014).

### Socioeconomic hardships and subsequent incidence of CVD

After adjustment for age (model 1), the recession-related socioeconomic hardships were associated with a higher incidence of CVD. The risk of CVD was 27% higher among women who experienced socioeconomic hardships as compared to those who did not (HR 1.27, 95% confidence interval (CI) 1.06–1.53, *P*=0.012; Table II).

**Table II.** Hazard ratios for CVD events according to the level of socioeconomic hardships caused by the 1990s economic recession.

Level of socioeconomic hardships due to the economic recession binaries			
Variables	Did not experience socioeconomic hardships (reference group) (*n*=331)	Experienced socioeconomic hardships (*n*=587)	*P* value
No. of cases of CVD, %	171 (51.7%)	330 (56.2%)	
HR model 1*		1.27 (1.06–1.54)	0.012
HR model 2*		1.26 (1.04–1.52)	0.017
HR model 3*		1.27 (1.05–1.53)	0.013
HR model 4*		1.25 (1.03–1.51)	0.021
HR model 5*		1.26 (1.05–1.52)	0.015
HR model 6*		1.26 (1.05–1.53)	0.015
HR model 7*		1.23 (1.02–1.50)	0.029

Values are hazards ratios (95% confidence intervals).

Model 1*: adjusted for age

Model 2*: adjusted for model 1 plus education, marital status.

Model 3*: adjusted for model 2 plus smoking status.

Model 4*: adjusted for model 3 plus alcohol intake.

Model 5*: adjusted for model 4 plus physical activity.

Model 6*: adjusted for model 5 plus vegetables, fruits and berries consumption.

Model 7*: adjusted for model 6 plus the history of IHD, T2D or hypertension.

CVD: cardiovascular disease; IHD: ischaemic heart disease; T2D: type 2 diabetes.

Further adjustments for SEP variables, the behavioural or lifestyle variables, and the prior history of IHD, T2D and hypertension did not affect the association. In model 2 (education and marital status), the risk of CVD was increased by 26% (HR 1.26, 95% CI 1.04–1.52, *P*=0.017).

After adjustment for smoking status in model 3, a 27% increased risk of CVD was still observed (HR 1.27, 95% CI 1.05–1.53, *P*=0.013).

Further adjustments for alcohol intake, physical activity and vegetables, fruits and berries consumption in each of the following models did not attenuate the association (HR 1.25, 95% CI 1.03–1.50, *P*=0.021; HR 1.26, 95% CI 1.05–1.53, *P*=0.015; and HR 1.26, 95% CI 1.05–1.53, *P*=0.015, respectively).

Finally, in model 7 (history of IHD, T2D and hypertension), the observed risk of incident CVD was increased by 23% (HR 1.23, 95% CI 1.02–1.5, *P*=0.029).

## Discussion

Our population-based prospective study among 918 women from Eastern Finland suggests an increased risk of incident CVD among women who reported having felt socioeconomic hardships during the severe economic recession which had occurred about 5 years prior to the start of the follow-up.

The impact of economic recessions on population health in general, and on CVD in particular, is still unclear and controversial. Reasons for this include the complexity of CVD determinants [1] and the limited literature on the topic [21].

Most studies on recessions and CVD have used unemployment as the only measure of socioeconomic hardship. For example, in Sweden between 1987 and 2003, which included the 1990s recession period, the unemployment rate was associated with higher CVD mortality among both genders [5, 6]. In Finland, a population-wide study on 2.5 million men and women aged 25–59 years demonstrated that individuals who became unemployed between 1987 and 1992 had higher all-cause mortality rates than those who stayed in their jobs [4]. Similar findings were observed in a longitudinal population-based study during the major economic recession in Brazil in 2014–2016. The remarkable increase in unemployment was linked to more than 30,000 additional deaths during the recession, mainly from CVD and cancer [22].

In our study, women who experienced hardships during the recession had more likely experienced unemployment at a younger age before the 1990s recession we studied. This suggests that unemployment may not have affected only women who lost their jobs during the recession, but also those who had no financial security and stable income. It is worth noting that recession-induced socioeconomic hardships among our study participants did not necessarily result from the women themselves losing their jobs. It also included reporting unemployment of the spouse or children. It is plausible that when the family is affected, women feel it also, at least on a psychological level.

Psychological factors such as depression, stress and anxiety are highly believed to deteriorate cardiovascular health during economic hardship times and over longer terms. The evidence on the plausible biological mechanisms linking psychosocial stress and CVD is still scarce. However, a number of mechanisms are suggested to contribute to atherosclerosis development and triggering of cardiovascular events. These include alterations in the autonomic nervous system, the inflammatory and neuro-hormonal functions, in addition to promoting unhealthy lifestyle behaviours [23]. For example, hypertension was suggested to result from stress and changes in working hours during the economic recession in Iceland in 2008, but only in men [24]. Whereas women demonstrated hypertension many years later [1]. Recently, the recession was found to be associated with an increase in IHD events among both genders [25].

Gerdtham and Ruhm (2006) found that each 1% reduction in the unemployment rate increased mortality by 0.4% when studying the effects of unemployment on CVD incidence during recessions in 23 Organisation for Economic Co-operation and Development (OECD) countries between 1960 and 1997 [9]. Ruhm further evaluated the fatality of CHD and acute myocardial infarction among men and women aged 35–85 years in the United States from 1972 to 1991 [7, 10]. Interestingly, the author concluded that the CVD mortality rate was lower during recessions, whereas a higher incidence of CVD was observed during a strong economy. This was explained by the higher prevalence of non-healthy behavioural risk factors of CVD such as smoking, obesity, physical inactivity and unhealthy diet during times of prosperity. These findings were supported by a German study conducted between 1980 and 2000 [8].

A study on the 2008 financial crisis in England on 9000 households between 2001 and 2013 showed a decrease in smoking and alcohol drinking, but also a decrease in fruit consumption during the recession. Obesity, diabetes and mental disorders continued to increase. Budget constraints had probably led to unhealthy dietary habits. These associations were observed especially in women. Unemployment during the recession had no different impact on health compared to the pre-recession period [26]. These findings raise the argument that the effects of recession on CVD lifestyle factors remain uncertain, and positive changes in specific behavioural risk factors do not necessarily lead to improvements in health status.

In our study, smoking seems to have some role in developing CVD among the women affected by the recession. Smoking could be a coping mechanism, although harmful, in the middle of the psychological distress caused by the recession.

Overall, there has been a growing interest in health research to study economic recessions and their impact on population health. The economically catastrophic COVID-19 pandemic will certainly trigger a surge of studies in this field [27]. Already earlier studies have shown that macroeconomic catastrophes and prolonged financial crises weaken health systems and expenditure on health [28]. More attention should be given to health policies and preparatory measures at all levels of society, keeping in mind that economic crises are recurrently expected to happen.

### Strengths of the study

The main strength of our study is the regionally representative population-based sample and the long follow-up time. The nationwide system of registers in Finland has been utilised in our epidemiological study. Therefore, our follow-up outcome measure can be considered reliable. The broad range of well-validated measures to adjust for can be added to the reliability of our findings.

Another strength is that most epidemiological studies focus on the role of socioeconomic position during the normal life setting, whereas little is known about the effect of massive economic hits such as recessions on population health. In addition, most of the previous studies on economic recessions have only used unemployment as the measure of experiencing socioeconomic hardships, while the detailed questionnaire used in our study draws a broader estimate on how the participants were overall affected by the recession, and not only by losing their jobs.

Moreover, our comprehensive dataset allows us to investigate a wide spectrum of CVD morbidity compared to most of the previous studies on recessions, which have mostly studied CVD mortality only, due to their data restrictions.

There is gender bias in the literature on socioeconomic hardships and CVD. This is in part also true with the KIHD study, which enrolled women later after it was first initiated to investigate the CVD risk factors in men. To date, still only about 8% of more than 600 KIHD-based international scientific articles have looked at women. Therefore, the core strength of the present research simply comes from the fact that it examines how subsequent CVD risk behaves in middle-aged and older women, who either had or had not been exposed to socioeconomic hardships during the recession.

### Limitations of the study

This study is based on an ethnically homogenic population of middle-aged and older Finnish women, which may limit the generalisability of our results to men and other ethnicities. However, results could be generalised to other Nordic countries that have similar demographic characteristics to Finland and follow the same type of social and welfare system. The results may also not be generalised to women of all ages.

Income and occupation were already implemented in the participants’ responses on whether they were affected by the recession or not. Therefore, we included only education and marital status in the SEP model as covariates to avoid over adjustment.

Finally, the study assesses the association between socioeconomic hardships and CVD, rather than the causal effect of the hardships on CVD.

## Conclusions

Our findings from Finland suggest that economic recessions may pose health risks to middle-aged and older women. Socioeconomic difficulties felt by the women themselves or their families during the 1990s economic severe recession in Finland were associated with an increased risk of developing CVD within a 20-year follow-up.
